# High Working Memory Capacity at the Cost of Precision?

**DOI:** 10.3390/brainsci9090210

**Published:** 2019-08-21

**Authors:** Anne-Katrin Vellage, Patrick Müller, Marlen Schmicker, Jens-Max Hopf, Notger G. Müller

**Affiliations:** 1 German Centre of Neurodegenerative Diseases, 39120 Magdeburg, Germany; 2 Berlin School of Mind and Brain, Humboldt-University, 10117 Berlin, Germany; 3 Leibniz-Institute for Neurobiology, 39120 Magdeburg, Germany; 4 Department of Neurology, Otto-von-Guericke University, 39120 Magdeburg, Germany; 5 Center for Behavioral Brain Sciences (CBBS), 39120 Magdeburg, Germany

**Keywords:** working memory capacity, precision, attention

## Abstract

Working memory capacity (WMC) varies tremendously among individuals. Here, we investigate the possibility that subjects with high WMC use this limited resource more efficiently by reducing the precision with which they store information in demanding tasks. Task difficulty was increased by (a) presenting more objects to be memorized, (b) informing subjects only after the encoding phase about the relevant objects, and (c) delivering distracting features at retrieval. Precision was assessed by means of a continuous delayed-estimation task, in which object features had to be estimated from memory. High WMC subjects did not show a stronger drop in precision in difficult tasks. Instead, a positive correlation between precision and general WMC emerged. These findings suggest that high WMC subjects do not necessarily trade in quantity for quality when forming working memory (WM) representations under increasing demand. Instead, they seem to be able to devote more cognitive resources to support WM storage.

## 1. Introduction

Working memory—the ability to maintain and manipulate information in memory for a short period of time—is a core cognitive function that is correlated with a number of cognitive skills such as reading comprehension [1], fluid intelligence [2], and reasoning ability [3]. Working memory capacity (WMC), that is, the number of items one can store in memory for a short time, varies considerably among humans, whereby some can only keep around two items in mind (low WMC), whereas others can store more than four (high WMC) [4].

It is currently unclear whether such high WMC subjects really have more memory resources (slots) available or whether they use their likewise limited resources more efficiently under increased memory load. In this regard, two major theories have been proposed: the fixed slots model [5,6,7,8,9,10], which assumes that, in a given individual, working memory capacity is limited to a fixed, small number of items; and the continuous or flexible resource model [2,11,12,13], which postulates that individuals can devote their resources flexibly to either a small number of items with precise and detailed representations or a large number with rather coarse representations. Whereas the first model would assume that subjects with a high capacity must have more resources or slots at their disposal, the latter model entails the possibility that these subject may increase their memory capacity by reducing the precision of the represented items in difficult situations (e.g., high load); in other words, high WMC subjects may trade in quantity for quality in such situations.

Support for the idea that high capacity subjects may make a more efficient use of their likewise limited resources comes from studies that related the capacity to store a large number of task-relevant items to the ability to filter out distracting, task-irrelevant information. These studies have shown that high WMC subjects are better at hindering irrelevant information from entering working memory (WM) [14]. On a neurophysiological level, it was observed that the so-called contralateral delay activity (CDA), an electrophysiological correlate of memory encoding, increased in amplitude in high WMC subjects only when the number of to-be-remembered target stimuli was increased. In low WMC subjects, the amplitude became larger also when irrelevant distractors were added to the display, suggesting that these subjects unnecessarily encoded the irrelevant information, too. Likewise, McNab & Klingberg [15] observed that WMC correlated positively with filtering efficiency, as indexed by neural activity in the frontobasal gatekeeper network of the brain. In sum, these studies suggest that high WM capacity is rather related to effective attentional selection than a large number of storage slots.

Another way to ‘boost’ WMC without providing more storage slots would be to trade in the number of representations with their precision according to the flexible resource theory [2,11,12,13]. Such a tradeoff would have been overlooked in many common tests of WMC, namely those relying on ‘change detection’ recognition tasks in order to calculate the capacity according to the formulas provided by Pashler [16] and Cowan [17]. In these tasks, the individual WMC is typically calculated from the performance on two-alternative forced choice tasks that require binary yes/no responses [8,18,19]. Hence, in this type of task, even imprecise representations of stored items usually lead to correct answers. 

In order to circumvent this pitfall, here we adopted an approach called continuous delayed-estimation [13,20] that allows us to separately assess WM precision and WM capacity. Instead of yes/no decisions, this task requires a quantification of the memory content on a continuous scale during retrieval, which is more informative about the quality of memory representations than binary decisions. For example, instead of just reporting whether or not a certain color is the same as in the previous display, subjects are required to match the color of the test stimulus to the memorized color as exactly as possible on a continuous color scale.

This approach allows to us test whether high WMC subjects indeed have more memory resources available or whether they use limited resources more effectively by reducing the precision of memory representations in demanding WM tasks. If the latter was true, then high WMC, in contrast to low WMC subjects, should produce disproportionately more errors in difficult (e.g., high load) than easy tasks (e.g., low load). In more demanding situations, their errors will then erroneously be more often based on non-target information and on guesses [21]. In contrast, the fixed slots model would be incompatible with such a tradeoff; instead, high WMC subjects would show superior memory, both in terms of quality (precision) and quantity (capacity), in both easy and difficult situations, as they generally have more resources (or slots) available to them [22].

The current study aims to answer whether the amount of available resources is comparable in high and low WMC subjects, while only their distribution differs between both groups, or whether high WMC subjects can provide more resources to form both multiple and precise representations. To that end, we assigned participants to high and low WMC based on the composite score of an independent WM test battery that comprised a wide range of different WM tasks in order to get a representative picture of their WM abilities. We then compared the continuously measured precision of the WM representations of low and high performing subjects in tasks of different difficult levels. Task difficulty was varied in three ways: set-size, timing of the cue, and the congruency of the non-memorized object feature. All of these manipulations have been shown to modulate task performance [4,23]. If their higher capacity was only based on a tradeoff between quality and quantity, while the amount of available resources was the same as in low WMC subjects, then the high WMC subjects should demonstrate disproportionally worse performance in the difficult variants of the task (i.e., a high difference score between high and low load, pre- and post-cue trials, and so on) and there should be a negative correlation between capacity and precision. If the high WMC subjects had more resources available, then a positive correlation and no specific performance drops in the difficult tasks should emerge. Note, however, that as outlined in more detail in the discussion, answering the question of whether or not high WM subjects can rely on more memory resources will not necessarily answer the old question of whether the resources for WM are generally fixed or flexible. 

## 2. Materials and Methods

Participants: A total of 20 young (11 females, 9 males, mean age 25.3 years, range 22–33 years), right-handed healthy participants with normal or corrected-to-normal vision were recruited from an academic environment and included in the analysis. They were paid volunteers and gave written informed consent before participation. All methods were carried out in accordance with relevant guidelines and regulations. The study was approved by the local ethics committee of Otto-von-Guericke University.

Subjects performed two sessions on two different days with one week in between. They started the first session with the WM precision test, which was followed by the first three subtests from the adapted paper and pencil WM capacity test (Lern und Gedächtnistest 3 (LGT-3) battery, Table 1). Half of the participants started with color stimuli and the other half with orientation stimuli in the working memory precision (WMP) test. On the second day, those subjects who had started with color stimuli were presented with orientation stimuli, and vice versa. Furthermore, on the second day, subjects performed the last three subtests from the WM capacity test.

### 2.1. Working Memory Capacity Test (LGT-3)

We used material from LGT-3 (Lern und Gedächtnistest 3 [27]) to assess the individual WMC. The test was chosen for three reasons. First, there was no overlap of this paper pencil test with our computerized precision test, which should avoid circularity when dividing subjects into low and high performers. Second, as numerous subtests using both verbal and figural material are taken into account to calculate the composite score used for group assignment, the test provides a broad estimate of different aspects of WM rather than assessing only one specific aspect, such as in typical span tasks. Third, the difficulty of the tests was adapted to young participants of the age of 16–35 years; thus, ceiling effects that might occur in more clinically oriented tests could be avoided. 

In our version of the task, the retrieval phase immediately followed the encoding phase of each subtest (otherwise, episodic memory may have been challenged, too). The first subtest was a route on a map that had to be memorized first and then had to be drawn on an empty map. In the second test, subjects had to learn word pair associations (German–Turkish, note that no participant spoke Turkish) and recognize them on a list of words. In the next subtest, subjects were presented with figural objects that had then to be reproduced verbally. On the second day, subjects were asked to memorize fictive phone numbers of fictive institutions and recall these numbers afterwards. In the following subtest, a report was read to the subjects and they had to recall facts such as numbers or names from this report. The last test was again a figural test, where subjects were asked to memorize fictive company signs and recognize them on a list of signs. 

### 2.2. Working Memory Precision Test

**Figure 1:**
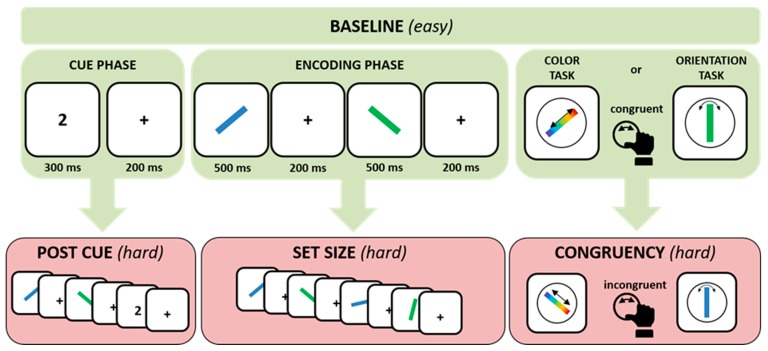
Schematic illustration of working memory precision tasks.

For the WM precision task, subjects had to memorize either the color or the orientation of a cued bar and adjust the color or orientation of a test bar at the end of the trial via a response dial. In the baseline (easy) version of the precision task, a pre-cue (number) indicated which of the following stimuli had to be memorized. After the cue, two displays with colored and tilted bars (study items) were shown one after the other. After a variable delay period, a test stimulus was presented and subjects used a dial to match the color or orientation to that of the memorized stimulus. Task difficulty was manipulated in three ways:

- Set size: Instead of two bars, subjects were presented with a series of four bars.
- Cue: The number cue was presented after the study items had been shown (post or retro cue), so that subjects were informed of which bar was relevant after the encoding phase.
- Congruency: Whereas in the easy version, the test bar had the same color (in orientation task) or orientation (in color tasks) as the studied bar (congruent), in the difficult version, the test bar’s non-relevant feature was one of the non-memorized study items (incongruent, see Figure 1). Note that there were also neutral trials in which the irrelevant feature of the test bar did not match to any of the preceding study items. However, these trials were not included in the present analysis. All these manipulations were intermixed, corresponding to a 2 × 2 × 2 design.

Stimuli: Bars were presented for 500 ms with delay periods of 200 ms in between, in which a fixation cross was presented. The cue was presented for 300 ms. The delay period lasted between 600 and 1200 ms. Color and orientation tasks were presented on different days, and trials with different set size, cue type, and congruency were presented in random order. In total, subjects had to perform 1464 trials.

Pre- and post-cues were numbers (0.8 × 0.6°) presented at the center of the screen. The colors of bars were randomly chosen from 180 colors drawn from a circle (radius 55°) in the CIE L*a*b color space, centered at L = 50, a = 17, and b = 6. The colors in one memory set had a minimum distance of 30°. Orientations were randomly chosen with a minimum distance of 15°.

### 2.3. Data Analysis

Firstly, the participants were grouped into low and high WMC based on the results of LGT-3. The percent of correct responses from each subtest was calculated and the mean of all subtests (composite score) was then used for a median split, by which subjects were separated into a group of high and low performers for further analyses. To analyze the performance, a repeated-measures analysis of variance (ANOVA), with the within factor subtest (subtest 1–6) and session order (day 1, day 2) as covariate, was run.

Secondly, working memory precision was assessed using a computer-based paradigm. The retrieval error in each trial was assessed by calculating the angular deviation between the target feature and the reported response feature, as done in previous studies [28,29]. As the present data were in a circular space, we used the definition of mean and standard deviation from Fisher [30]. We then analyzed the general effects of task difficulty on precision errors (standard deviations) using a mixed-design ANOVA with the within subject factors set size (2/4), cue (pre-cue, post-cue), and congruency (congruent, incongruent) and the between subject factor group (high WMC, low WMC). Additionally, in order to assess their errors in more detail, we assessed whether high WMC subjects may produce more errors based on non-targets and guesses in difficult tasks; we divided errors into three different components (proportion target response, PT; proportion non-target responses, PNT; proportion uniform responses, PU), following Bays et al. [31] and analyzed the data using ANOVA.

Thirdly, to assess our main question more directly, that is, whether high WMC subjects produce less precise responses in difficult tasks, we calculated difference scores, by subtracting standard deviations in the easy conditions from those in the hard conditions (set size 4–set size 2; post-cue–pre-cue; incongruent–congruent). On the basis of the difference scores, we ran a multivariate ANOVA. 

Last, in order to assess the general relation between memory precision and capacity, measures of WM capacity (LGT mean performance) and overall precision (mean standard deviation, collapsed over all conditions of the experimental manipulations) were entered into a bivariate correlation analysis. 

## 3. Results

### 3.1. Working Memory Capacity (LGT-3)

A repeated-measures ANOVA with the within factor subtest (subtest 1–6) and session order (day 1/2) as covariate revealed no significant main effect of subtest (F(3,62) = 2.390, *p* = 0.070, Greenhouse–Geisser corrected), so performance of the subtests was averaged to a mean performance score, which was 66.40 (± 2.21 SEM). 

### 3.2. Working Memory Precision—General Effects

**Figure 2:**
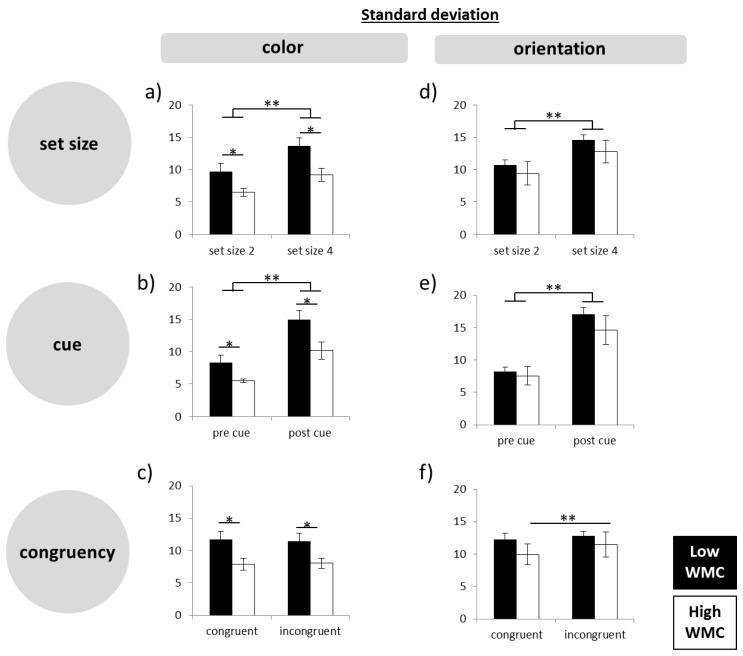
Standard deviations from the precision task for color and orientation separated for each task condition (set size, cue, congruency). The three manipulations of task difficulty led to more imprecise responses. WMC, working memory capacity. ∗= *p* ≤ 0.05; ∗∗= *p* ≤ 0.01.

In a first analysis, we checked whether the task difficulty manipulations had the predicted effects (Figure 2). The mixed-design ANOVA for the color task showed a main effect of set size (F(1,18) = 63.414; *p* < 0.001) and cue (F(1,18) = 60.649; *p* < 0.001), but no effect of congruency (F(1,18) = 0.070; *p* = 0.795). Similarly, in the orientation task, significant effects were found for set size (F(1,18) = 27.327; *p* < 0.001), cue (F(1,18) = 56.212; *p* < 0.001), and a trend for the congruency condition (F(1,18) = 4.258; *p* = 0.054). In general, high WMC subjects showed superior performance, indicated by a main effect of group in the color task (F(1,18) = 6.169, *p* = 0.023) and a trend towards significance in the orientation task (F(1,18) = 3.691, *p* = 0.071).

### 3.3. Working Memory Precision—Error Components

**Figure 3:**
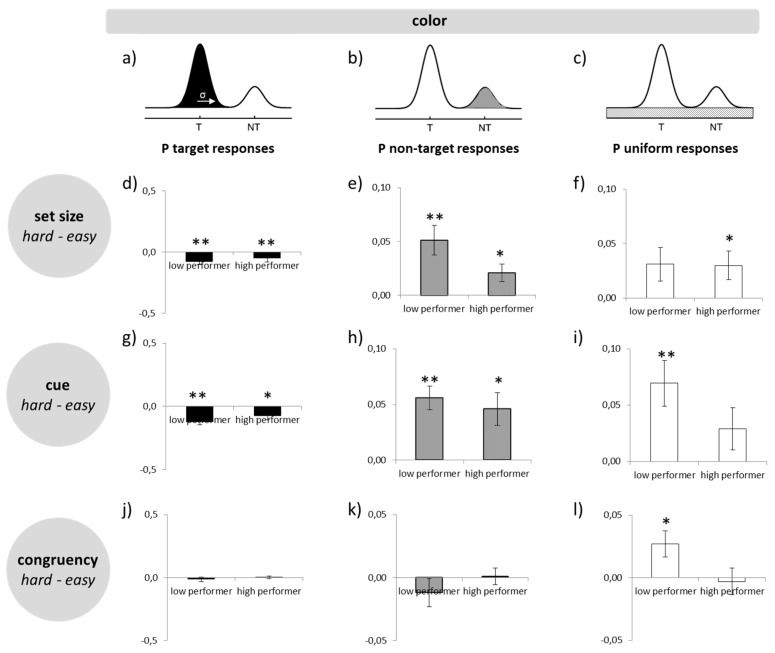
Distribution of errors from the precision task for orientation and color: (**a**–**c**) schematic illustration of error contributions; (**d**–**f**) difference between set size 2 and set size 4 separated by performance group and error source; (**g**–**i**) difference between pre-cue and post-cue separated by performance group and error source; (**j**–**I**) difference between incongruent and congruent separated by performance group and error source. ∗ = *p* ≤ 0.05; ∗∗ = *p* ≤ 0.01.

An ANOVA comparing the difference scores of the three error types (PT, PNT, PU) did not reveal any group difference (all *p* > 0.05; Figure 3). In other words, high WMC subjects in difficult tasks did not start to make more guesses or based their responses more often on wrong objects.

### 3.4. Working Memory Precision—Difference Scores 

**Figure 4:**
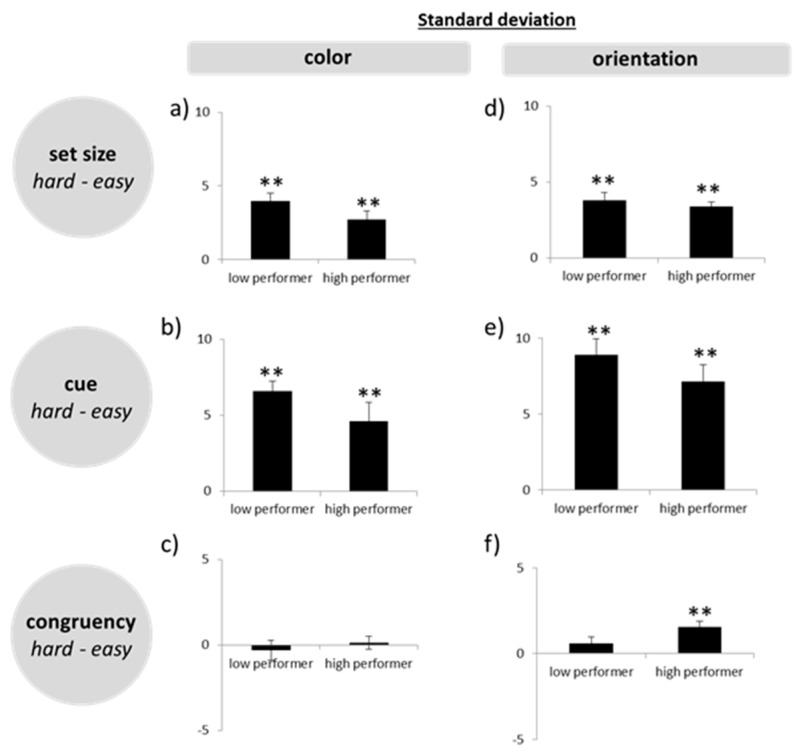
Difficulty scores (standard deviations easy–hard) from the precision task for color (**a**–**c**) and orientation (**d**–**f**) separated for each task condition (set size, cue, congruency). ∗∗ = *p* ≤ 0.01.

A multivariate ANOVA on the difference scores in both the color and the orientation task did not reveal group differences for set size, cue, and response type (all *p* > 0.1, see Figure 4). Hence, high WMC subjects did not show a disproportionate performance drop in the more difficult tasks.

### 3.5. Correlation Analysis

**Figure 5:**
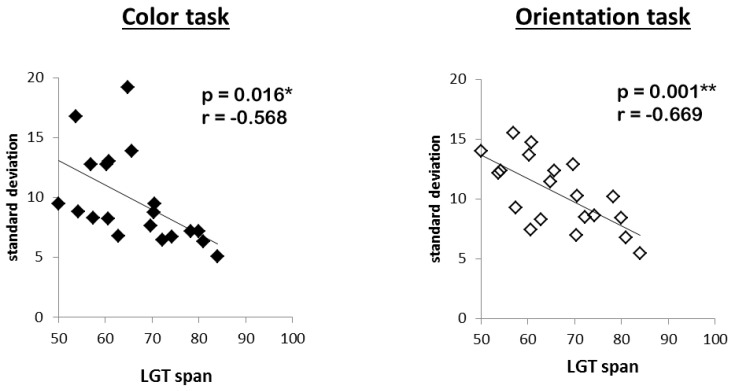
Statistical relations between working memory precision for color and orientation and working memory capacity (Lern und Gedächtnistest (LGT) performance). ∗ = *p* ≤ 0.05. ∗∗ = *p* ≤ 0.01.

The correlation analysis revealed significant negative correlations between precision errors of memory for color and LGT performance (r(20) = –0.568, *p* = 0.016) and between memory precision for orientation and LGT performance (r(20) = –0.669, *p* = 0.001). In other words, subjects with high WMC gave more precise responses than subjects with low WMC (Figure 5).

## 4. Discussion

In the present experiments, easy–hard manipulations of different quality were used in order to shed light on the question of how high performing subjects may achieve their higher WM capacity. The manipulations were as follows: small versus large set size, pre-cue versus post-cue, and congruent versus incongruent retrieval. The combination of these variations induced different levels of difficulty with trials involving four objects, post-cues, and incongruent answers constituting the most difficult level. In line with the intended experimental manipulation and in line with the literature (e.g., the works of [12,31] for a review), we found that precision was lower when four instead of two objects had to be stored in memory. Of note, this was only the case when post-cues were applied. In the case of pre-cues, subjects could always direct their attention to a single object, namely the one whose position within the sequence corresponded to the preceding cue number. They could ignore the other, task-irrelevant objects, rendering the representation of the relevant (cued) object less susceptible to interference. As a consequence, precise memory representations could be formed for the cued object. With post-cues, on the other hand, attention could be directed to the relevant object only after all objects had been presented. This means that no study object could be ignored, as with pre-cues; instead, all objects had to be encoded to some extent first. Post-cues, nevertheless, have been shown to induce better performance compared with no-cue conditions, indicating that attention can also modulate memory representations after the initial encoding has taken place [32,33]. In spite of this, with post-cues and especially in the high load condition, the initial encoding process was more challenging than in pre-cue trials, where subjects could focus on less stimulus information. Nevertheless, like in the other difficult conditions, high WMC subjects did not reduce precision in this task more than low WMC subjects.

Regarding our initial question, the reported findings clearly speak against the assumption that subjects with high WMC must rely on the same limited amount of resources as low WMC subjects, and that they achieve their higher capacity only by sacrificing details of the stored items in more difficult tasks. High WMC subjects did neither reduce the precision with which they reported target features in demanding situations (high load, post-cue, distracters present), nor did they base their responses more often on the wrong objects, nor did they make more guesses. Instead, their performance remained superior to that of low WMC subjects even in these demanding situations. Accordingly, we found a negative correlation between precision errors and WMC, indicating that high WMC subjects not only stored more objects in memory, but did so with higher acuity. Hence, the results support the idea that high WMC subjects do have access to more resources when storing visual information in WM (general capacity view [22]).

Regarding an old debate in the WM scientific community, at first sight, the present findings seem to support the fixed slot model, not the flexible resource model, as for the latter, a tradeoff between quality and quantity constitutes a crucial hallmark. However, at a closer look, the situation is less unequivocal. *Had* the high WMC subjects showed a tradeoff, then the situation would have been clear, as only a flexible model would have been reconcilable with this finding. The fact, however, that no such tradeoff emerged and, instead, these subjects outperformed the low WMC subjects in all conditions, including the more difficult ones, is only consistent with a model that assumes high performing subjects can allocate more cognitive resources to the WM task at hand. Whether these resources are flexible or fixed cannot be unambiguously answered, though. Even a model of flexible resources can be reconciled with precise representations in difficult tasks—as long as a sufficient amount of resources is at the disposal of the high performing subjects. Hence, the lack of a tradeoff between precision and WMC in our high performing subjects could reflect the fact that even our difficult tasks did not push these young and healthy subjects to their limits, so they just did not need to trade in preciseness for capacity. In other words, even more difficult tasks may be required for a tradeoff to emerge. Although this possibility cannot be ruled out completely based on the present data, there is some evidence speaking against this assumption. In accordance with previous observations [34], not only was there no correlation between capacity and precision in the present study, instead there was a positive one. In other words, the more items one can store, the more precisely he/she does so. This is especially remarkable as we assessed WMC in a set of tasks that was completely unrelated to the precision task. It is not very probable that such a pattern would emerge should a quality/quantity tradeoff represent a crucial mechanism for increasing one’s WM capacity. Instead, the observation that WMC positively correlated with precision in our experimental task speaks to the idea that WM constitutes a core process related to general cognitive functioning. This does not necessarily mean that high WMC subjects “possess” more resources, they might instead use them more efficiently, for example, by limiting the resources unnecessarily devoted to other, task-irrelevant processes [35]. It also demonstrates that WM capacity and WM precision seem to be based on very similar processes in the visuospatial domain, independent of task. 

### Limitations

Apart from the already mentioned possibility, namely that even our difficult tasks did not challenge the high WMC subjects enough for a tradeoff between capacity and precision to emerge, there are some other limitations that need to be considered. First, the sample size was small (*n* = 20), with all participants recruited from an academic environment. Next, Fougnie et al. [21] found that the quality of visual working memory varies considerably across trials within an individual. This observation offers an alternative account for the observed performance differences that is somewhat beyond the scope of this report. Finally, we deliberately chose to use a rather uncommon test to determine WMC. This may make our results less comparable to others who use traditional span tasks for this purpose. Such tasks, however, in our view are not representative for real life situations and are too similar to the utilized precision task, which is why we used other tests to determine WMC. 

## 5. Conclusions

Good WM performance has been shown to be crucial for scoring high in exams [36] and in real-world situations, whereas low WM performance is found in many clinical populations [37]. Our current results underline the assumption that WM capacity is indeed related to the amount of cognitive resources an individual can devote to task relevant processes, which recommends WMC as a good proxy for general task solution abilities.

## Figures and Tables

**Table 1:** Overview of experimental procedure. WMP, working memory precision; LGT-3, Lern und Gedächtnistest 3.

SUBJECTS	TESTS	DAY 1	DAY 2
***N* = 10**	**WMP Task**	Color	Orientation
	**LGT-3**	Test 1–3	Test 4–6
***N* = 10**	**WMP Task**	Orientation	Color
	**LGT-3**	Test 1–3	Test 4–6

Presentation: All computer-based tests were generated using the Psychophysics Toolbox [24,25,26] and Matlab 2013a (The MathWorks, Inc., Natick, Massachusetts, United States).
